# Characteristic Fluctuations of Dynamic Power Delay Induced by Random Nanosized Titanium Nitride Grains and the Aspect Ratio Effect of Gate-All-Around Nanowire CMOS Devices and Circuits

**DOI:** 10.3390/ma12091492

**Published:** 2019-05-08

**Authors:** Yiming Li, Chieh-Yang Chen, Min-Hui Chuang, Pei-Jung Chao

**Affiliations:** Parallel and Scientific Computing Laboratory, Department of Electrical and Computer Engineering, National Chiao Tung University, Hsinchu 300, Taiwan; cychen@mail.ymlab.org (C.-Y.C.); mhchuang@mail.ymlab.org (M.-H.C.); pjchao@mail.ymlab.org (P.-J.C.)

**Keywords:** DC and AC characteristic fluctuations, gate-all-around, nanowire, MOSFETs, work function fluctuation, aspect ratio of channel cross-section, timing fluctuation, noise margin fluctuation, power fluctuation, CMOS circuit, statistical device simulation

## Abstract

In this study, we investigate direct current (DC)/alternating current (AC) characteristic variability induced by work function fluctuation (WKF) with respect to different nanosized metal grains and the variation of aspect ratios (ARs) of channel cross-sections on a 10 nm gate gate-all-around (GAA) nanowire (NW) metal–oxide–semiconductor field-effect transistor (MOSFET) device. The associated timing and power fluctuations of the GAA NW complementary metal–oxide–semiconductor (CMOS) circuits are further estimated and analyzed simultaneously. The experimentally validated device and circuit simulation running on a parallel computing system are intensively performed while considering the effects of WKF and various ARs to access the device’s nominal and fluctuated characteristics. To provide the best accuracy of simulation, we herein calibrate the simulation results and experimental data by adjusting the fitting parameters of the mobility model. Transfer characteristics, dynamic timing, and power consumption of the tested circuit are calculated using a mixed device–circuit simulation technique. The timing fluctuation mainly follows the trend of the variation of threshold voltage. The fluctuation terms of power consumption comprising static, short-circuit, and dynamic powers are governed by the trend that the larger the grain size, the larger the fluctuation.

## 1. Introduction

The dimension of effective devices has shrunk to a sub-22 nanometer scale, and due to this, we are facing even more serious characteristic variability problems [1,2,3,4,5,6,7]. High-κ/metal gate (HKMG) technology has been recognized as a solution to solve intrinsic fluctuation, but the crystal orientation of nanosized metal grain is uncontrollable during the growth step under high temperatures [8,9]. Values of uncertain orientation-dependent work functions (WKs) of gate material causes WK fluctuation (WKF). Many studies have surveyed WKF for different devices [3,10,11,12,13,14,15,16], and some have further discussed the distribution of metal grains on planar metal–oxide–semiconductor field-effect transistors (MOSFETs) [17,18]. However, seldom do these studies put emphasis on gate-all-around (GAA) nanowire (NW) MOSFET devices. As a result, in this study, we will focus on estimating the impact of WKF on the electrical characteristics of GAA NW MOSFET devices and its implication for the dynamic property of a complementary metal–oxide–semiconductor (CMOS) circuit. In order to analyze WKF, we apply the newly developed localized WKF (LWKF) method [18]. The averaged work function method (AWKF) [19] was reported to estimate the entire value of WKs, but the process of averaging cannot be used to estimate the effect of local WKs on device characteristics. The physically-sound LWKF method is an effective technique that can determine the random number and random location effects, as well as the physical phenomena of the localization of nanosized metal grains, and it does not underestimate the effect of WKF [20]. Hence, we adopt the LWKF method to explore the WKF effect. According to the properties of the metal material, TiN has two different orientations: <200> and <111>, with 60% and 40% generated probabilities [19,21,22]. In addition to WKF, the limitation of process fabrication may lead to geometrical variations in channel cross-sections and affect the operations of devices. Due to this, perfectly round-shaped channel GAA NW MOSFET devices are difficult to manufacture. The different aspect ratio (AR) of channel radius results in a different shape of channel cross-section—an elliptical shape instead of the ideal round shape [23,24,25]. Therefore, we will discuss the electrical characteristics of the explored devices with different ARs. Additionally, we will simulate the combination effect of WKF and the variation of AR. WKF-induced circuit variations, such as timing and power fluctuations [26,27,28], seriously affect the dynamic property of GAA NW CMOS circuits. Most previous research only focused on the DC characteristics of N-type planar or fin-typed MOSFET devices when considering the aforementioned variability [10,28,29,30]. Various fluctuations of circuit characteristics, such as noise margin (NM), timing, and power consumption are also important to research, but the variability of GAA NW CMOS circuits has not been clearly studied yet. To comprehensively explore the aforementioned issues for 10 nm gate GAA NW MOSFETs and CMOS circuits induced by WKF and different ARs, we extend an experimentally-calibrated three-dimensional (3D) quantum-mechanically-corrected device and circuit simulation [1,18,26,27,28,29]. The engineering findings of this study indicate that falling time (t_f_) is lower than rising time (t_r_) owing to the relatively larger driving capability of the N-type device. Along with the increasing grain number of higher WKs, the high-to-low delay time (t_HL_) and the low level of noise margin (NM_L_) become higher, while the low-to-high delay time (t_LH_) and the high level of noise margin (NM_H_) decrease. All power consumption terms follow the trend that the larger the grain size, the larger the fluctuation.

This paper is organized as follows: In Section 2, we describe the statistical device simulation techniques of WKF and AR for the GAA NW MOSFETs and CMOS circuits. In Section 3, we discuss and analyze the simulation results of WKF combined with the variation of AR on GAA NW MOSFETs and CMOS circuits. Finally, we draw the conclusions of this study and suggest future work.

## 2. Statistical LWKF and AR Simulation Techniques

**Figure 1:**
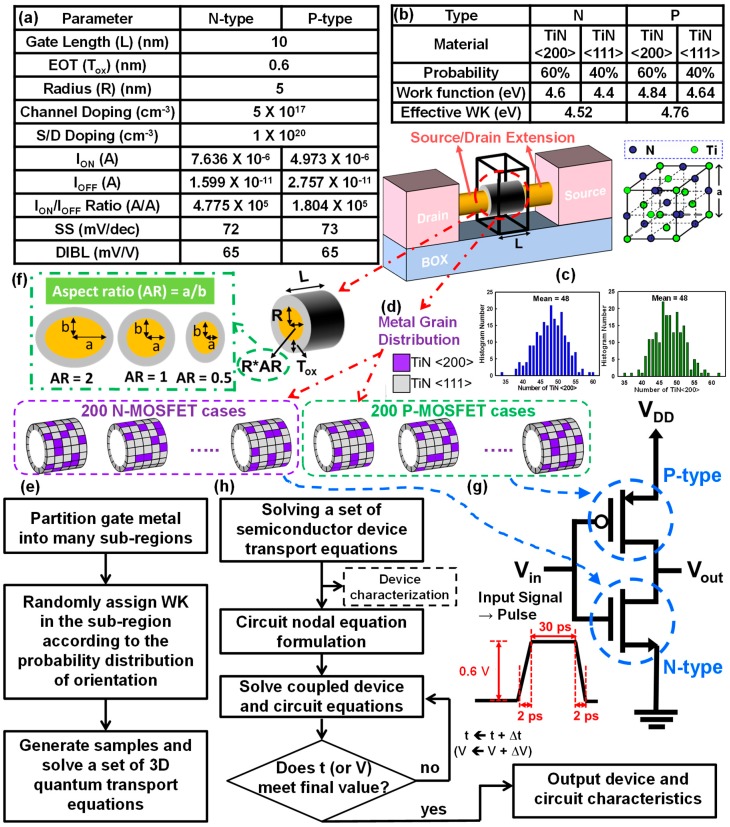
(**a**) The device’s parameters and the nominal short-channel effect (SCE) values of the N-/P-type devices. We used TiN, which is a stable compound with a NaCl (sodium chloride) structure as the metal gate. According to the properties of the metal material, TiN has two different orientations: <200> and <111>, with 60% and 40% generated probabilities [19,21,22]. The related work functions (WKs) of the N-/P-type devices are shown in (**b**). (**c**) A histogram plot of the number of high WKs generated following Gaussian distribution. (**d**) The adopted test device with low and high WKs, in which the light color and dark color represent the low and high WKs, respectively. (**e**) The flow chart of work function fluctuation (WKF) simulation, where 200 cases for the N-/P-type devices were generated and simulated, as shown in (**d**). (**f**) The illustration and definition of different aspect ratio (AR) devices. (**g**) The tested complementary metal–oxide–semiconductor (CMOS) inverter circuits in this study. (**h**) Simulation flowchart for the coupled device–circuit approach. MOSFET: metal–oxide–semiconductor field-effect transistors; S: source; D: drain; DIBL: drain-induced barrier lowering; EOT: effective oxide thickness; SS: subthreshold swing; 3D: three-dimensional.

In this work, we extended the statistical device simulation technique [3,31] to analyze WKF and different ARs of GAA NW CMOS circuits. Figure 1a shows the device setting parameters, the device characteristics, and the achieved nominal values of the short-channel effect (SCE) of the studied N-/P-type devices. To conduct the simulation and to estimate the impacts of WKF, we used the LWKF method for statistical device simulation, which is illustrated in Figure 1b–e in detail. We used TiN as the metal gate material, which includes two different orientations: <200> and <111> with the associated 60% and 40% probabilities. The related parameters are shown in Figure 1b. To calibrate the magnitude of threshold voltage (V_th_) to 280 mV, we used the WK-tuning techniques in which the metal gate is doped by hydrogen plasma/fluorine ion implantation, as this was found by K. Han et al. [32,33] to achieve different WK values. Thus, the corresponding WKs are 4.6 and 4.84 eV and 4.4 and 4.64 eV, respectively, for the N-/P-type devices. First, to carry out the WKF simulation, we partitioned the TiN metal gate of the GAA NW MOSFET devices into many sub-regions according to grain size. Second, Figure 1c shows a histogram plot of the number of high WKs, which were generated according to Gaussian distribution. Then, the high and low WKs were randomly assigned and mapped onto the sub-region of the gate region of device, as shown in Figure 1d. Finally, we acquired the statistically generated surface for WKF simulation. For the N-/P-type devices, 200 cases were generated and simulated, as shown in Figure 1d, where the regions of light color and dark color represent the low and high WKs, respectively. Figure 1e is a flow chart of the LWKF simulation. The illustration and definition of different AR devices are given in Figure 1f. The device channel has major axis “a” and minor axis “b” of different lengths of channel radius. The AR is defined as the ratio of the length of the major axis to that of the minor axis, which equals “a/b”. The length of the minor axis of the ellipse-shaped channel is fixed at 5 nm, and the major axis varies with an AR of 0.5, 1, and 2, respectively, in our simulation setting. To discuss and analyze the variations that experience both WKF and variation of AR, we used a new extension of the LWKF method for the explored device with respect to different ARs [18,20] that can be implemented in device simulation. We utilized the CMOS inverter circuit consisting of N- and P-type GAA NW MOSFETs as the tested circuit to explore the timing and power fluctuations induced by WKF and the effect of AR. The schematic plot of the GAA NW CMOS inverter circuit is shown in Figure 1g. The logic input signals of the N- and P-type GAA NW MOSFETs were “1” to “0” and “0” to “1”. The transition time, including rising delay time, as well as the falling delay time and the hold time of the input signal were 2, 2, and 30 ps, respectively. To estimate and capture the influence of WKF on the circuit characteristics of the explored GAA NW CMOS inverter, a coupled device–circuit simulation approach was employed, as shown in Figure 1h. This was used because a well-established equivalent circuit model of GAA NW CMOS devices is still unavailable. At first, an initial guess for device bias was assumed, and the device characteristics in the test circuit were estimated by solving the device transport equations. The obtained result was the initial guess for the coupled device–circuit simulation. Then, based on Kirchhoff’s current law, the nodal equations of the tested circuits were formulated. Because the device equations were solved in the coupled device–circuit simulation, the effects of WKF on the device and the CMOS inverter circuit characteristics were thus properly captured. The coupled simulation was solved iteratively until the solution converged in each time step and bias condition.

To validate our simulation, we examined the band profile along the channel by solving 3D quantum mechanical transport and non-equilibrium Green’s function models. Then, we calibrated the simulation result with measurement data of the fabricated sample [34,35]. For both the N- and P-type devices, the I_D_–V_G_ characteristics of the simulated device at V_D_ = 1/−1 V were experimentally calibrated to the measured data by fitting the mobility model parameters [18,20,34,35]. Because the I_D_–V_G_ characteristics are well-fitted between the fabrication and the simulation, this further ensures the accuracy of our statistical device and circuit simulation.

## 3. Results and Discussion

**Figure 2:**
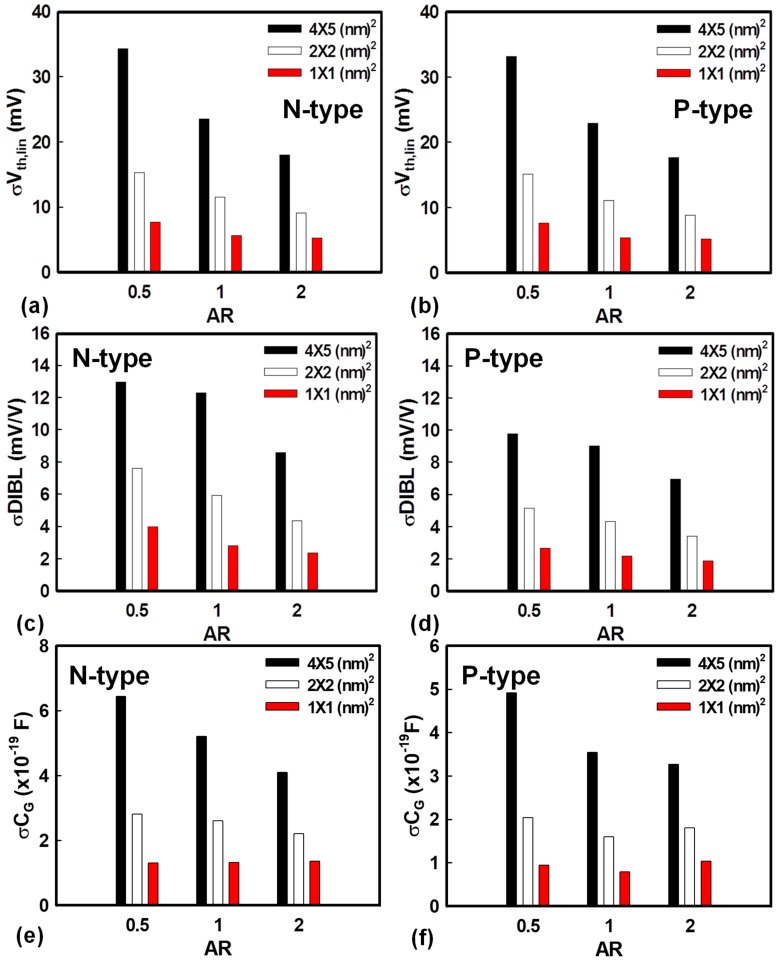
The standard deviation of (**a**), (**b**) V_th_, (**c**), (**d**) DIBL, and (**e**), (**f**) gate capacitance (CG) versus different ARs with respect to different grain sizes of N-/P-type gate-all-around (GAA) nanowire (NW) MOSFETs affected by WKF. For both the N- and P-type GAA NW MOSFETs, devices with a larger grain size and smaller AR have greater deviations than the others.

Figure 2 shows the standard deviation (σ) of threshold voltage, drain-induced barrier lowering (DIBL), and gate capacitance (C_G_) versus AR with respect to different grain sizes of N- and P-type GAA NW MOSFETs. As the grain size reduced from 4 × 5 to 1 × 1 nm^2^ and the AR induced from 0.5 to 2, σV_th_ reduced, as shown in Figure 2a,b. For a fixed channel area with a different grain size, if the grain size is large, the same gate area may contain only a few grains, so the effective WKF will be governed by high or low WKs and further lead to higher or lower V_th_, causing relatively larger variation. Under the condition of the same grain size, the device with the larger AR has smaller fluctuation, because the grain size is relatively small. As shown in Figure 2c,d, the case of AR = 0.5 had the highest deviation, indicating that the device with the critical dimension is more sensitive to variation in the process. According to the definition of DIBL, the magnitude of σDIBL in Figure 2c,d had a similar trend to σV_th_, as shown in Figure 2a,b, due to the dependency on V_th_. Figure 2e,f shows the bar charts of σC_G_ with three different ARs and three different grain sizes. The devices with a larger AR had a larger surface area, so the value of C_G_ with a larger AR was larger than that of the smaller AR. However, under the condition of the same grain size, the larger AR had the smaller fluctuation. This is because the area of AR = 2 was larger, and the grain size was relatively small. Thus, the magnitude of σC_G_ of larger AR devices was smaller. Notably, the aspect ratio was given from a fixed axis, so it would also be helpful to interpret the result versus the device dimension using the plot of a Pelgrom model. Although we have applied the Pelgrom model to explore the variability of fin-type field-effect transistors (FinFETs) [36], the same model, we assumed, can be applied to examine the variability of a GAA NW MOSFET device.

**Figure 3:**
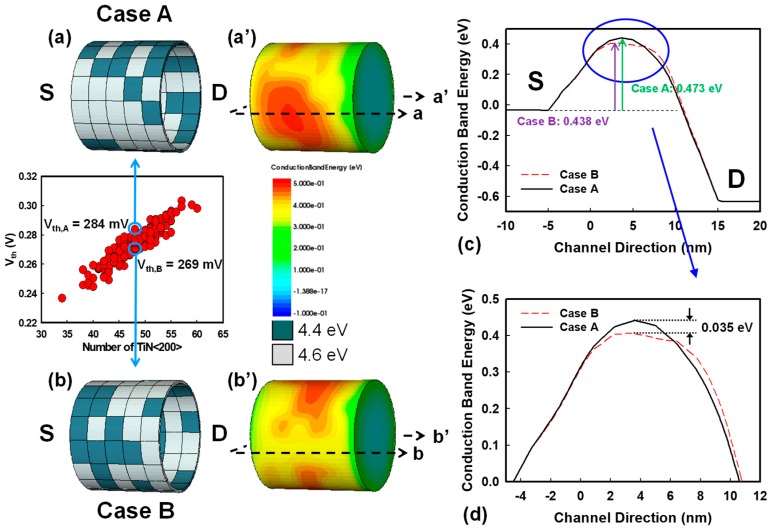
(**a**,**b**) The metal grain distribution of Case A and Case B that have the same number of high WKs but different V_th_. For the metal gate, the green color represents low WKs, and the white color indicates high WKs. (**a’**,**b’**) The corresponding conduction band energy distributions in the channel region. The bias conditions are V_D_ = 0.6 V and V_G_ = 0 V. (**c**) The one-dimensional (1D) conduction band energy profile, and (**d**) the zoom-in plot from the source side to the drain side of Case A and Case B. Case A has a higher barrier in the off-state, which leads to higher V_th_ compared with Case B.

Figure 3 shows the effects of a random number and random position of high WK grains on the threshold voltage: threshold voltage increases when the number of high WK grains increases. Notably, the charge distribution is strongly governed by different WKs locally. By using the LWKF method, we determined the random location effect and found that most of the high WK grains are near source (S) side or drain (D) side. Figure 3a,b shows the distributions of Case A and Case B with the highest and lowest V_th_ in the group of the same number of high WKs, respectively. The green color represents low WKs, and the white color indicates high WKs. Figure 3a’,b’ shows the corresponding conduction band energy distributions in the off-state. Because the grain pattern of Case A has a larger proportion of high WKs near the source side compared with Case B, in order to explore the difference, we illustrate the one-dimensional (1D) conduction band energy profile of the device channel center in Figure 3c. Figure 3d is a zoom-in plot and the black solid line and the red dashed line represent Case A and Case B, respectively. The barrier of Case A is 35 meV higher than that of Case B. Thus, the case with the higher barrier needs a higher voltage to lower the high barrier and make the electrons easier to pass through, leading to higher V_th_.

**Figure 4:**
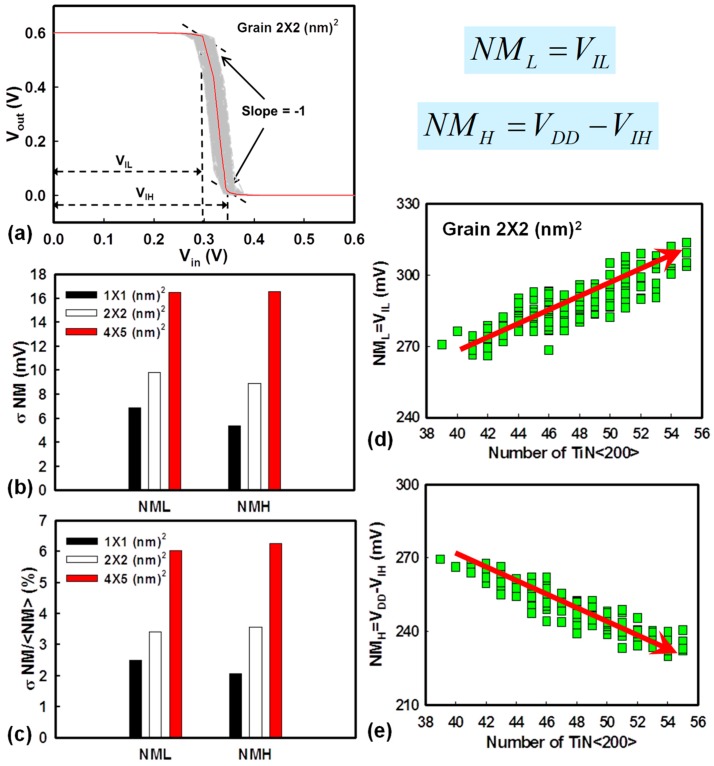
(**a**) The voltage transfer curves of NW MOSFET inverter circuits fluctuated by WKF. V_IL_ and V_IH_ are used to determine the noise margin (NM) of the inverter. The slope at these two points of voltage transfer curves is −1V/V. The related definitions of low level of noise margin (NML) and high level of noise margin (NMH) are shown below (**a**). (**b**,**c**) Plots of the fluctuated NM induced by the WKF of a GAA NW MOSFET with three grain sizes: 4 × 5 nm^2^ (red), 2 × 2 nm^2^ (white), and 1 × 1 nm^2^ (black), respectively. (**b**) The standard deviation and (**c**) the coefficient of variance of NM. (**d**,**e**) Plots of NML and NMH versus the number of TiN <200> with grain size equal to 2 × 2 nm^2^. The trend of NM versus high WK number between NM_L_ and NM_H_ acts conversely. When the high WK number increases, NM_L_ also increases, and thus, NM_H_ decreases.

**Figure 5:**
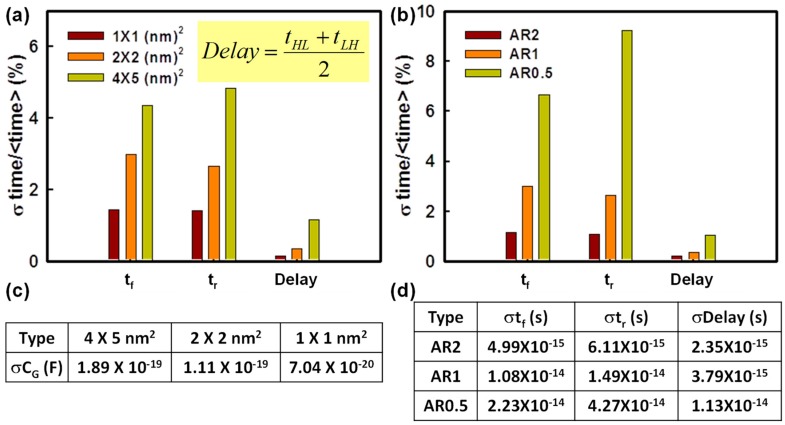
(**a**,**b**) The coefficient of variance of the timing analysis of GAA NW circuits experiencing WKF with different grain sizes and different ARs, respectively. (**c**) The fluctuation of load gate capacitance with three different grain sizes. (**d**) The associated values of fluctuated timing parameters of different ARs. The bias condition is V_D_ = 0.05 V and V_G_ = 0.6 V.

**Figure 6:**
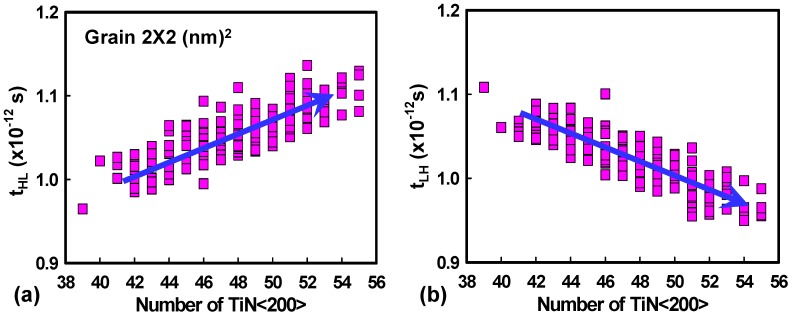
(**a**,**b**) The high-to-low delay time and low-to-high delay time versus the number of TiN <200> with grain size equal to 2 × 2 nm^2^. With the increasing number of high WKs, the high-to-low delay time has become higher, and thus, the low-to-high delay time has become lower.

**Figure 7:**
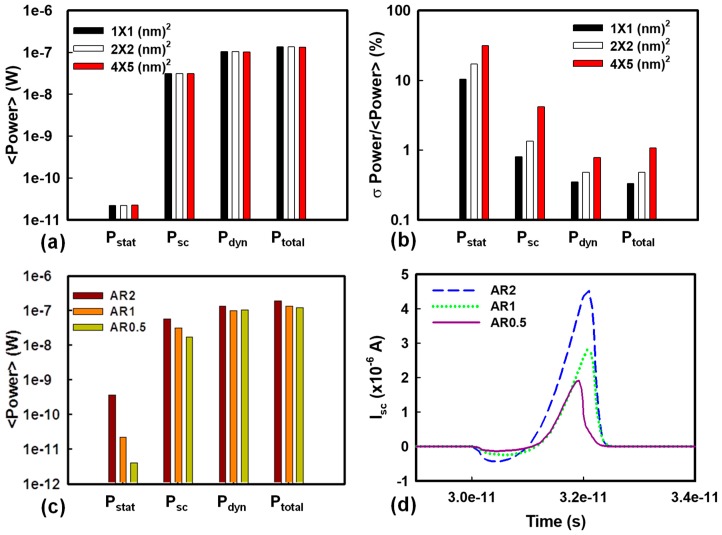
(**a**,**b**) Plots of each fluctuated power consumption experiencing WKF of the tested circuit with different grain sizes. (**a**) The average and (**b**) the coefficient of variance of power consumption. (**c**) The average power consumption induced by WKF of the tested circuit with different ARs. (**d**) The short-circuit current of the circuit with AR2 (blue dashed line), AR1 (green dotted line), and AR0.5 (purple solid line), respectively.

Figure 4a shows the fluctuated voltage transfer curves induced by the WKF of the explored CMOS inverter circuit. V_IL_, the maximum permitted logic “0” at input, and V_IH_, the minimum permitted logic “1” at input, are the extracted input voltages of the voltage transfer curves at the slope of −1V/V. These two points are used to determine NM_H_ and NM_L_. The definition of NM is shown in Figure 4. The values of NM_H_ and NM_L_ are indicators to estimate the maximum noise signal tolerance during the operation of the inverter circuits. Figure 4b,c shows the bar chart of NM, which increases with an increasing grain size, similar to the variation of V_th_ in Figure 2a,b. Hence, NM also follows the trend of σV_th_. Figure 4d,e displays the plots of NM_L_ and NM_H_ versus the number of high WKs affected by WKF with grain size fixed at 2 × 2 nm^2^. When the number of high WK metals increases, NML rises and NMH does the opposite. Higher WK numbers cause a higher value of N-type V_th_ and a lower value of P-type V_th_, resulting in both the values of V_IL_ and V_IH_ becoming higher. This leads to an increasing NM_L_ and a decreasing NM_H_. Figure 5 shows the variance of the timing of the tested circuits experiencing WKF with three different grain sizes and three different ARs. The magnitude of variance of t_f_ is smaller than that of t_r_ owing to the larger driving capability of the N-type device. The device with the larger driving capability requires less time to charge/discharge the load capacitance. Hence, it exhibits less fall time fluctuation. The “Delay” is defined as the average of t_HL_ and t_LH_. The larger the grain size, the larger the fluctuation of the delay time. This can be explained by the load capacitance fluctuation in Figure 5c. The σC_G_ of the grain equal to 4 × 5 nm^2^ is the largest among the three different sizes of metal grains. A larger σC_G_ would lead to a longer σDelay. The associated values of the timing fluctuation of different ARs are given in Figure 5d, which can verify the trend in Figure 5b—the larger the AR, the larger the timing fluctuation. Figure 6 shows t_HL_ and t_LH_ versus the number of high WKs fluctuated by WKF with grain size equal to 2 × 2 nm^2^. The trend of t_HL_ increases when the number of high WK metals increases, because the delay time is dependent on the start of the signal transition, which indicates the magnitude of V_th_. Along with the rising high WK number, the value of the N-type V_th_ increases, and it becomes harder for the N-type device to turn on, causing a higher t_HL_. For P-type devices, a larger number of high WKs leads to a lower value of V_th_, so t_LH_ decreases. Figure 7 shows the related results of the power consumption affected by WKF and various ARs of the tested circuit.

The total power (*P_total_*) is composed of static power (*P_stat_*), short-circuit power (*P_sc_*), and dynamic power (*P_dyn_*). The definitions of these power components are as follows:

(1) $$P_{stat}=\;V_{DD}I_{leakage}$$

(2) $$P_{sc}=\;f_{0\to 1}V_{DD}\int_{T}I_{sc}\left(\tau \right)d\tau$$

(3) $$P_{dyn}=\;C_{load}V_{DD}^{2}f_{0\to 1}$$

(4) $$P_{total}=\;P_{stat}+P_{sc}+P_{dyn}$$

where *I_leakage_* is the leakage current that flows between the power rails when operating at static state. *f*_0→1_ is the clock rate. *I_sc_* is the short-circuit current, which is observed when both the N- and P-type devices are turned on simultaneously, resulting in a DC path between the power rails. *T* is the switching period. *P_stat_* will consume as long as the *V_DD_* is opened, regardless of the switching activity between input and output. *P_sc_* is determined by *I_sc_* and the time of existence of the DC path between the power rails. *P_dyn_* is determined by the load capacitance (*C_load_*).

Figure 7a,b shows the bar chart of power consumptions of different grain sizes. In Figure 7a, it can be observed that the average values of *P_sc_* and *P_dyn_* were the dominating roles in power dissipation. As shown in Figure 7b, all the power consumption terms followed the trend that the larger the grain size, the larger the fluctuation. For *P_dyn_*, the device with grain size equal to 4 × 5 nm^2^ displayed larger *P_dyn_* owing to its larger *C_load_* compared with the others. The device with grain size equal to 1 × 1 nm^2^ had smaller *P_stat_* than the devices with the other two grain sizes, because *I_leakage_* of 1 × 1 nm^2^ was the smallest of the grain sizes. Additionally, Figure 7b shows that the magnitude of the variance of *P_stat_* was the largest among all power consumption terms. However, its contribution to *P_total_* was marginal. As a result, *P_total_* was mainly affected by *P_sc_* and *P_dyn_*. Figure 7c shows the average power dissipation affected by the WKF of different ARs. The average values of *P_sc_* and *P_dyn_* were also much larger than that of *P_stat_*. Therefore, for all AR devices, *P_sc_* and *P_dyn_* were the dominating factors in *P_total_*. In addition, *I_sc_* in the case of AR = 2 was the largest in Figure 7d, and this shows that devices with AR = 2 had the largest *P_sc_*.

## 4. Conclusions

In this work, DC/AC characteristic fluctuation of GAA NW MOSFETs and variation of the dynamic property of a CMOS circuit induced by WKF and ARs of channel cross-sections were investigated using an experimentally calibrated 3D device and circuit simulation running on a parallel computing system. The V_th_ diminished with a decrease in grain size for both the N- and P-type devices. DIBL followed the trend of V_th_ due to the dependency on the V_th_ of DIBL. The standard deviation of C_G_ with large grain size also had greater fluctuated value. We conclude that for both DC and AC characteristics, the smaller the grain size, the lower the fluctuation. The threshold voltage increases when the number of high WK grains increases. For devices with the same number of WKs, the device with a larger proportion of high WKs near the source side will achieve to a higher threshold voltage. In addition, under the condition of same metal grain size, the larger AR device has a less severe impact from WKF than a smaller AR device, because it has a large effective gate area and the grain size is relatively small. Hence, larger AR devices will average the effect of random metal grain fluctuation and thereby reduce the degradation of WKF. For the variation of the dynamic property of the explored CMOS circuit, the delay time and NM fluctuations follow the trend of V_th_—that a larger variation is caused by a larger grain size. For t_f_ and t_r_, the larger driving capability of the N-type device is the reason t_f_ is smaller than tr. NML is positively related to the number of high WKs, while NMH is negatively related to it. In power dissipation, both P_sc_ and P_dyn_ are the most significant fluctuation sources.
